# Characterization of the Endometrial MSC Marker Ectonucleoside Triphosphate Diphosphohydrolase-2 (NTPDase2/CD39L1) in Low- and High-Grade Endometrial Carcinomas: Loss of Stromal Expression in the Invasive Phenotypes

**DOI:** 10.3390/jpm11050331

**Published:** 2021-04-22

**Authors:** Aitor Rodríguez-Martínez, Carla Trapero, August Vidal, Josep Maria Piulats, Inmaculada Gómez de Aranda, Jean Sévigny, Maria Eulàlia Fernández-Montolí, Jordi Ponce, Xavier Matias-Guiu, Mireia Martín-Satué

**Affiliations:** 1 Departament de Patologia i Terapèutica Experimental, Facultat de Medicina i Ciències de la Salut, Campus Bellvitge, Universitat de Barcelona, 08907 L’Hospitalet de Llobregat, Spain; arodriguezm@ub.edu (A.R.-M.); ctrapero@ub.edu (C.T.); avidal@bellvitgehospital.cat (A.V.); igomezdearanda@ub.edu (I.G.d.A.); xmatias@idibell.cat (X.M.-G.); 2 Oncobell Program, Institut d’Investigació Biomèdica de Bellvitge (IDIBELL), CIBERONC, 08908 L’Hospitalet de Llobregat, Spain; jmpiulats@iconcologia.net (J.M.P.); mefernandez@bellvitgehospital.cat (M.E.F.-M.); jponce@bellvitgehospital.cat (J.P.); 3 Servei d’Anatomia Patològica, Hospital Universitari de Bellvitge, 08907 L’Hospitalet de Llobregat, Spain; 4 Servei d’Oncologia Mèdica, Institut Català d’Oncologia (ICO), 08908 L’Hospitalet de Llobregat, Spain; 5 Centre du Recherche du CHU de Québec-Université Laval, Québec City, QC G1V 4G2, Canada; jean.sevigny@crchudequebec.ulaval.ca; 6 Départment de Microbiologie-Infectiologie et d’Immunologie, Faculté de Médecine, Université Laval, Québec City, QC G1V 4G2, Canada; 7 Servei de Ginecologia, Hospital Universitari de Bellvitge, 08907 L’Hospitalet de Llobregat, Spain

**Keywords:** NTPDase2, endometrial carcinoma, adenomyosis, purinergic signaling, ATP, CD39, endometrial MSC, SUSD2

## Abstract

Ectonucleoside triphosphate diphosphohydrolase-2 (NTPDase2/CD39L1) has been described in human non-pathological endometrium in both epithelial and stromal components without changes along the cycle. It was identified as a stromal marker of basalis. In the present study, we aimed to evaluate NTPDase2 distribution, using immunolabeling and in situ enzyme activity approaches, in endometrial carcinoma (EC) at different tumor grades. NTPDase2 was present in tumor epithelial EC cells, as in the non-pathological endometria, but the expression underwent changes in subcellular distribution and also tended to decrease with the tumor grade. In stroma, NTPDase2 was identified exclusively at the tumor-myometrial junction but this expression was lost in tumors of invasive phenotype. We have also identified in EC samples the presence of the perivascular population of endometrial mesenchymal stem cells (eMSCs) positive for sushi domain containing 2 (SUSD2) and for NTPDase2, already described in non-tumoral endometrium. Our results point to NTPDase2 as a histopathological marker of tumor invasion in EC, with diagnostic relevance especially in cases of EC coexisting with other endometrial disorders, such as adenomyosis, which occasionally hampers the assessment of tumor invasion parameters.

## 1. Introduction

The human endometrium, the mucous membrane lining the cavity of the uterus, consists of two layers: the functionalis, adjacent to the uterine cavity, which contains the surface epithelium, the glandular epithelium, and a substantial amount of vascularized stroma; and the basalis, adjacent to the myometrium, made up of the basal portions of the glands, a dense stroma, and blood vessels. The endometrium is a dynamic tissue with recurring cycles of regeneration to restore the functional layer, shed during menses. This regenerative capacity requires the presence of adult stem or progenitor cells. It has been suggested that stem/progenitor cells reside in the remaining basalis of the endometrium [1,2]. Clonogenic cells, or colony-forming units, have been identified in purified populations of human epithelial and stromal cells [3]. Despite the robust studies conducted in the field, nicely reviewed by de Miguel-Gómez et al. [4], more studies are required to deepen our knowledge of the regenerative capacity of the basalis. This highlights the need for describing endometrial basal stromal markers to provide tools for further study.

Extracellular adenosine triphosphate (ATP), its derived nucleotides, and the nucleoside adenosine are mediators of so-called purinergic signaling, which is involved in a wide range of physiological and pathological conditions, including proliferation, differentiation, motility, migration, death, and immune responses of cells, among others [5,6]. Ectonucleotide triphosphate diphosphohydrolase-2 (NTPDase2/CD39L1), a cell-surface member of the NTPDase (CD39) family of ectoenzymes, hydrolyses mainly ATP and, to a much lesser extent, adenosine diphosphate (ADP) [5]. We have previously described the expression of NTPDase2 in the cyclic and atrophic human endometrium without changes of expression along the menstrual cycle [7,8]. NTPDase2 is present in both surface and glandular epithelium although confined to cilia of ciliated cells [7]. Importantly, NTPDase2 has also been identified as a marker of human endometrial basal stroma, useful to trace diseases originating in the basalis, such as adenomyosis, defined by the presence of normal endometrial glandular and stromal cells in the myometrium [7]. Moreover, NTPDase2 is also expressed in the perivascular population of endometrial mesenchymal stem cells (eMSCs) positive for sushi domain containing 2 (SUSD2), the most abundant population of MSCs described in the human endometrium, located in both functionalis and basalis [7,9]. This finding coincides with a number of studies that revealed the implication of purinergic signaling in the regulation of the proliferation, differentiation, motility, migration, death, and immunomodulatory capacity of MSCs [10,11,12,13,14]. SUSD2^+^/NTPDase2^+^ perivascular cells have also been identified in the myometrium [7]. The presence of NTPDase2 in basal stromal cells and in eMSCs, along with the role of purinergic signaling in MSCs, suggests that NTPDase2 may be involved in the control of endometrial regeneration. The fine regulation of endometrial regeneration is essential for correct reproductive function, and it is necessary to avoid an uncontrolled proliferation leading to pathological states such as cancer.

Endometrial carcinoma (EC) represents the most common gynecologic cancer in women in Western populations, reaching 12% of cancer cases [15]. Classically, ECs are classified according to their histological characteristics into endometrioid endometrial carcinomas (EECs), comprising low-grade (grade 1 and grade 2), with better prognosis, and high-grade (grade 3) tumors; and the high-grade non-endometrioid carcinomas, including the serous carcinomas as the main type, but also others such as clear cell carcinomas, endometrial carcinosarcomas, and mixed carcinomas, mostly aggressive and with a poor prognosis [16,17]. As mentioned above, purinergic signaling is involved in a wide range of cellular processes, and the purinergic imbalance observed in some inflammatory and neoplastic processes evidences its involvement in tumorigenesis and cancer progression [18]; moreover, increased levels of extracellular ATP are found in tumors [19,20]. Different studies have confirmed the involvement of purinergic signaling in ECs. The membrane pore-forming ATP receptor P2X7 is downregulated in endometrial and other gynecological cancers, probably preventing P2X7-mediated apoptosis in these tumor cells [21,22,23,24]. Other ATP receptors, such as P2X4, have been identified in EC [25]. We have previously demonstrated the expression of the so-called CD39-CD73 axis in EC, with high ATPase (and ADPase) activity in part attributable to NTPDase1 (formerly CD39), highly expressed in the tumor stroma [26]. Ecto-5′nucleotidase (CD73), also highly expressed in tumor stroma, is able to complete the hydrolysis of the adenosine monophosphate (AMP) generated by CD39 to adenosine [26]. However, CD73 expression in epithelial tumor cells in EC decreases with the tumor grade and it has been identified as a possible modulator of epithelial migration and invasion in EC [27]. The expression of other ectonucleotidases contributing to the high nucleotidase activity in EC has not yet been studied, although their expression is well documented in non-pathological endometrium [28].

In the present study, we aimed to decipher the NTPDase2 expression in endometrial cancer at different tumor grades. We evaluated NTPDase2 expression at the stroma of the endometrial–myometrial junction both in non-invasive and desmoplastic invasive ECs, to determine whether this expression is related to the myofibroblast-like phenotype acquired by some stromal cells in EC. The chance to analyze EC samples coexistent with non-tumoral lesions, such as adenomyosis and endometrial polyps, allowed us to compare the stroma of the tumor with the non-tumoral stroma of the samples. Furthermore, we studied the presence, in EC, of the SUSD2^+^/NTPDase2^+^ eMSC population, already identified in non-tumoral endometrium.

## 2. Materials and Methods

### 2.1. Samples

The ethical principles of this study adhere to the Helsinki Declaration, and all the procedures were approved by the ethics committee for clinical investigation of Bellvitge University Hospital (PR179/18). Informed consent was obtained from all subjects involved in the study. Fifty-seven human EC samples were obtained from hysterectomy specimens and were diagnosed and classified by histology type and grading at the Pathology Service of Bellvitge University Hospital. Tumors included in the study were EECs (82.4%), serous carcinomas (8.8%), carcinosarcomas (5.3%), and mixed carcinomas (3.5%). The presence or absence of the following histological features of the recruited tumor cases was evaluated: 1) adenomyotic lesions, defined by the presence of endometrial glands and well-defined stroma within the myometrium away from the endometrial-myometrial junction, 2) invasion of the tumor into the myometrium, and 3) the presence or absence of surrounding reactive and fibrotic stroma, known as desmoplasia or desmoplastic reaction. Moreover, tumors were classified according to histological grading into three categories: I) high-grade tumors, including EECs grade 3 and non-endometroid carcinomas (47.4% of cases); II) medium-grade tumors, including EECs grade 2 (17.5%); and III) low-grade tumors, including EECs grade 1 (35.1%). Two cases presented endometrial polyps with EC. A descriptive statistical summary of tumor cases is included in Table 1.

For immunolabeling experiments, tissue samples were cut and fixed with 4% paraformaldehyde and soaked in 30% sucrose solution at 4 °C for 24 h for cryoprotection. Then, they were embedded in O.C.T. freezing medium (Tissue-Tek^®^; Sakura Finetk, Zoeterwoude, The Netherlands). Fifteen-μm sections were obtained using a Leica CM1950 Cryostat (Leica, Wetzlar, Germany). Sections were put onto poly-L-lysine coated glass slides and stored at −20 °C until use. Unless otherwise indicated, the reagents used were purchased from Sigma-Aldrich (Saint Louis, MO, USA).

### 2.2. Antibodies

The primary antibodies and their respective dilutions for immunohistochemistry and immunofluorescence experiments were mouse anti-NTPDase2 (Clone H9s; http://ectonucleotidases-ab.com) at 1 µg/mL, rabbit anti-NTPDase2 (CD39L1) (ALX-215-045, Enzo Life Sciences, Farmingdale, NY, USA) at 1:100, rabbit anti-SUSD2 (ab121214, Abcam, Cambridge, UK) at 1:400, and rabbit anti-alpha smooth muscle actin (α-SMA) (ab5694, Abcam, Cambridge, UK) at 1:200. Dilutions were made in phosphate-buffered saline (PBS). Secondary horseradish peroxidase (HRP)-conjugated ready-to-use goat anti-mouse and anti-rabbit antibodies (EnVision™ + System; DAKO, Carpinteria, CA, USA) were used for immunohistochemistry. Secondary goat anti-mouse Alexa Fluor 488 and goat anti-rabbit Alexa Fluor 555 (Life Technologies, Carlsbad, CA, USA) were used at 1:500, diluted in PBS, for immunofluorescence experiments.

### 2.3. Immunolabeling Experiments

Immunolabeling experiments were performed as previously described [7,8]. Tissue sections were washed twice with PBS to remove the O.C.T freezing medium. Non-specific binding of antibodies was blocked by pre-incubation of samples for 1 h at room temperature (RT) with PBS containing 20% normal goat serum (NGS, Gibco, Paisley, UK), 0.2% Triton, and 0.2% gelatin (Merck, Darmstadt, Germany). For immunohistochemistry experiments, previous blocking of endogenous peroxidase activity was performed with 10% methanol (*v*/*v*) and 2% H_2_O_2_ (*v*/*v*) in PBS for 30 min. Slices were then incubated overnight at 4 °C with the selected primary antibody. After three washes in PBS, tissue sections were incubated with the appropriate secondary antibody for 30 min in the case of HRP-conjugated antibodies and for 1 h in the case of fluorescence at RT. Secondary antibodies alone were routinely included as controls for the experiments.

For immunohistochemistry, the peroxidase reaction was performed in a solution containing 0.6 mg/mL 3, 3′-diaminobenzidine substrate (DAB; D-5637, Sigma-Aldrich, St. Louis, MO, USA) and 0.5 µL/mL H_2_O_2_ in PBS for 10 min and stopped with PBS. Nuclei were counterstained with haematoxylin and slides were then dehydrated and mounted with DPX mounting medium (BDH Laboratories, Dubai, UAE). Samples were observed under light Nikon Eclipse E200 (Nikon, Tokyo, Japan) and photographed under a light Leica DMD 108 microscope (Leica, Wetzlar, Germany). In fluorescence assays, for nucleus labeling, sections were mounted with aqueous mounting medium with DAPI (ProLong™ Gold antifade reagent with DAPI, Life Technologies, Paisley, UK). Samples were observed and photographed under a Zeiss LSM 880 Confocal Laser Scanning Microscope (Zeiss, Oberkochen, Germany). Fluorescence images were processed with the ZEN 2.3 SP1 software (Zeiss, Oberkochen, Germany).

Immunohistochemical staining was independently evaluated by three observers. Staining distribution was recorded as protein presence or absence in the selected tumor location.

### 2.4. In Situ ATPase Activity Experiments

In situ ATPase activity was detected on tumor sections using a protocol based on the Wachstein/Meisel lead phosphate method [8,29,30]. Frozen tumor sections were kept at RT for 10 min, washed twice with 50 mM Tris-maleate buffer pH 7.4, and pre-incubated for 30 min at RT with 50 mM Tris-maleate buffer pH 7.4 containing 2 mM MgCl_2_ and 250 mM sucrose. Enzymatic reaction was performed for 1 h at 37 °C in a buffer containing 50 mM Tris-maleate pH 7.4, 250 mM sucrose, 3% (*v*/*v*) dextran, 2 mM MgCl_2_, 2 mM CaCl_2_, 5 mM MnCl_2_, 2 mM Pb (NO_3_)_2_, and 2.5 mM levamisole, as an inhibitor of alkaline phosphatases, in the presence of 1 mM ATP as a substrate. Control assays were performed in the absence of ATP.

Released inorganic phosphate was revealed by incubation with 1% (NH_4_)_2_S (*v*/*v*) for exactly 1 min. Nuclei were counterstained with hematoxylin. Samples were mounted with Fluoromount™ (Sigma-Aldrich, Saint Louis, MO, USA), observed under light Nikon Eclipse E200 microscope, and photographed under a light Leica DMD 108 microscope.

### 2.5. In Silico TCGA (The Cancer Genome Atlas) and Survival Analysis

Data used for the in silico analysis correspond to the uterine corpus endometrial carcinoma (TCGA-UCEC), extracted from the The Cancer Genome Atlas (TCGA) through cBioPortal for Cancer Genomics (http://cbioportal.org). A total of 527 samples were used with integrated expression and clinical data. Gene expression data were downloaded as normalized fragments per kilobase of exon per million fragments mapped (FPKM) values, and Log2 transformed. Groups were compared with a one-way analysis of variance (ANOVA) test. Survival analysis was performed with R survminer package (created by Alboukadel Kassambara). For overall survival (OS) and progression-free survival/disease-free survival (PFS/DFS) curves, the median values were used as cut-off for ’High‘ and ’Low‘ groups. The results are displayed as Kaplan–Meier plots, and Log-rank test was used for comparison of the survival curves. A *p*-value < 0.05 is considered statistically significant.

### 2.6. Statistical Analysis of Ectonucleotide Triphosphate Diphosphohydrolase-2 (NTPDase2) Presence

The predictive analytics software IBM SPSS Statistics v22 (IBM Corp., Armonk, NY, USA) was used for the creation of frequency tables with the presence or absence and the distribution of NTPDase2 in each tumor component as well as for grading and invasion features of EC.

## 3. Results

### 3.1. Expression of NTPDase2 in Tumor and Stromal Cells in Endometrial Carcinomas (ECs)

**Figure 1:**
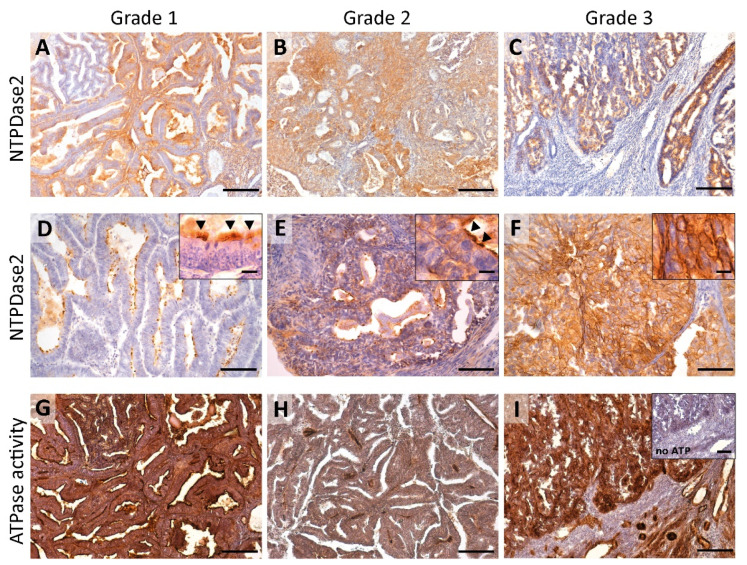
Immunohistochemistry α-NTPDase2 (ectonucleotide triphosphate diphosphohydrolase-2) (**A**–**F**) and in situ enzyme ATPase activity (**G**–**I**) in ECs at different tumor grades (Grade 1, 2, 3). NTPDase2 expression was mainly observed in the tumor epithelial cells of ECs (**A**–**F**). For stroma labelling, please refer to Figures 3 and 7. Grade 1 ECs showed apical NTPDase2 label, in cilia of tumor ciliated epithelial cells (inset in **D**; arrowheads). In Grade 2 tumors, NTPDase2 was also mainly detected apically, usually in cilia (inset in **E**; arrowheads). Grade 3 tumors displayed a strong NTPDase2 label in the whole cell (**F**). In situ ATPase activity was strongly detected as dark brown deposits with a high level of coincidence with NTPDase2 immunodetection (**G**–**I**). Inset in (**I**) corresponds to the activity experiment performed in the absence of substrate. Scale bars are 200 µm (**A**–**C**), (**G**–**I**), inset in **(I**), 100 µm (**D**–**F**), 25 µm for insets in (**D**–**F**).

We measured the expression of NTPDase2 in ECs through immunohistochemical experiments with two different antibodies (Enzo Life Sciences and ectonucleotidases-ab.com). The presence of NTPDase2 was detected in the epithelium, the stroma, and some perivascular cells of the tumor (Figure 1). The results were the same with the two anti-NTPDase2 antibodies. ATPase activity showed a high level of coincidence with the immunohistochemical results of NTPDase2, although other members of the family present in the tissue, such as NTPDase1, might also account for this activity (Figure 1).

#### 3.1.1. Redistribution of NTPDase2 Expression with the Tumor Grade: From Containment to the Cilia to the Whole Tumor Cell

**Figure 2:**
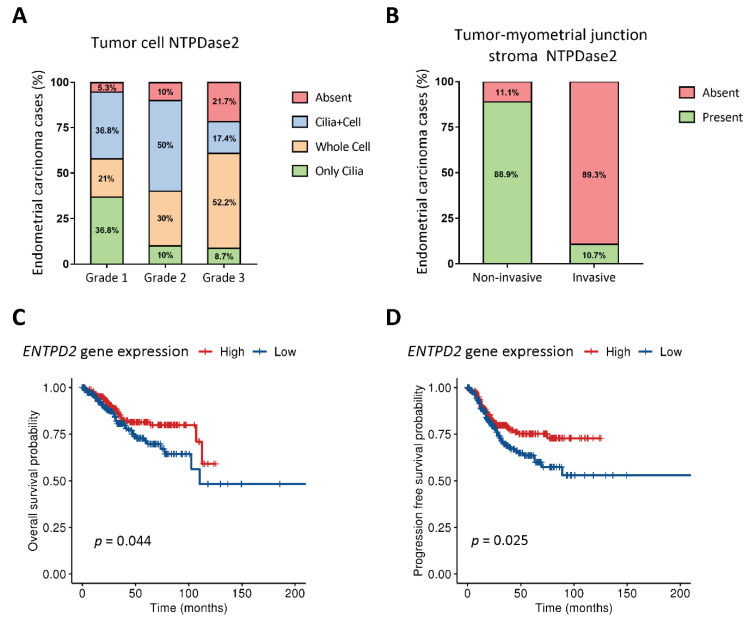
Percentage of EC cases with different pattern of NTPDase2 expression in tumoral epithelium (**A**) and in the stroma at the tumor-myometrial junctional zone (**B**). Low-grade tumors showed NTPDase2 expression mainly in cilia, while high-grade tumors lost this expression but increased the whole-cell distribution (**A**). NTPDase2 expression in grade 2 ECs was simultaneously present in both locations in 50% of cases (**A**). NTPDase2 labelling in the tumor-myometrial junction stroma was observed in most non-invasive EC cases, whereas invasive EC cases mostly lacked NTPDase2 (**B**). Kaplan–Meier curves of overall survival (OS) (**C**) and progression-free survival/disease-free survival (PFS/DFS) (**D**) probabilities extracted from The Cancer Genome Atlas uterine corpus endometrial carcinoma (TCGA-UCEC) cohort regarding *ENTPD2* gene expression showed a better clinical prognosis in high NTPDase2- bearing endometrial carcinomas. Log-rank test was used for comparison of the survival curves. OS: overall survival; PFS/DFS: progression-free survival/disease-free survival.

NTPDase2 was present in epithelial tumor cells but the number of tumors expressing the protein tended to decrease with the tumor grade; while 95% of grade 1 tumors expressed the protein, the percentage decreased to 78% in grade 3 tumors. However, the striking observation was the change in the subcellular distribution (Figure 2A). NTPDase2 was found in two, non-exclusive tumor cell localizations: the cilia of ciliated tumor cells (along the entire length of cilia) and throughout the whole cell (Figure 1). NTPDase2 expression was mainly detected in ciliated cells in low-grade ECs, as reported in non-tumor endometria, whereas the expression profile was generalized to the whole tumor cell membrane in high-grade ECs, a distribution not seen in non-tumor endometria. Intermediate patterns were found in grade 2. Percentages of each case are indicated in Figure 2A.

#### 3.1.2. NTPDase2 Expression in the Stromal Component of Tumor: Changes during Tumor Progression

In the stroma, NTPDase2 expression was restricted to the transitional limit between the endometrial zone and the myometrium, known as the endometrial-myometrial junction. Interestingly, this expression varied depending on the tumor’s invasive phenotype.

**Figure 3:**
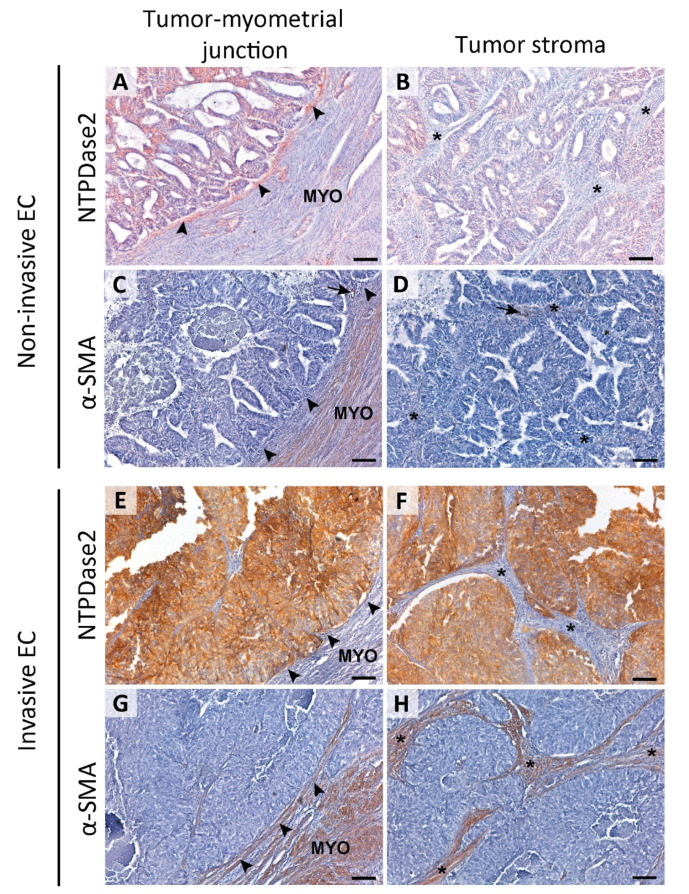
Immunolocalization of NTPDase2 and α-SMA in the stroma of non-invasive (**A**–**D**) and invasive ECs (**E**–**H**). NTPDase2 was immunodetected in the stromal cells located in the tumor-myometrial junction (**A**; arrowheads) of non-invasive EC, but not in the rest of the stroma (**B**; * asterisks). In these tumors, α-SMA expression was not detected either in tumor-myometrial junction stroma (**C**; arrowheads) or in the rest of the stroma (**D**; * asterisks), except in pericytes enwrapping some vessels (**C**,**D**; arrows). In invasive EC, the absence of NTPDase2 expression in stroma was generalized in the tumor-myometrial junction (**E**; arrowheads) as well as in the rest of the stroma (**F**; * asterisks). Conversely, the immunodetection of α-SMA was extended to tumor-myometrial junction (**G**; arrowheads) as well as to the rest of the stroma (**H**; * asterisks). Scale bars are 100 µm. MYO: myometrium.

Histological evaluation of the 57 ECs revealed 48 cases presenting invasive tumor glands in the myometrium and 9 cases with non-invasive patterns. In those 9 non-invasive tumors, we observed stromal NTPDase2 expression in endometrial-myometrial junction in 88.9% of cases (Figure 3A), all except one which was a grade 3 EEC. On the other hand, we confirmed that the myofibroblast marker α-SMA was absent in stromal cells of non-invasive ECs, except in pericytes (Figure 3C,D). In contrast, 89.3% of invasive tumors did not present NTPDase2 label in the stroma near or in the myometrium (Figure 3E,F). The other 10.7% of cases had the stroma labeled. Most of these invasive EC cases showed α-SMA expression in the stroma (Figure 3G,H). NTPDase2 relative expression results are represented in Figure 2B.

**Figure 4:**
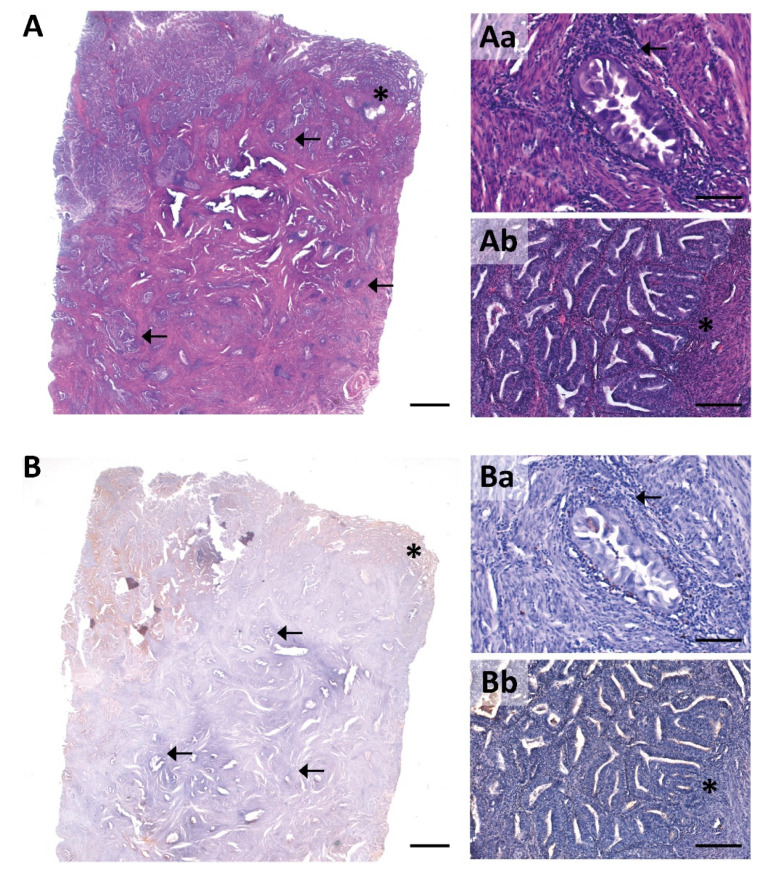
Consecutive sections of an invasive EC with haematoxylin and eosin staining (**A**) and NTPDase2 immunolabeling (**B**). Desmoplasia in the myometrium appeared as a reactive stromal tissue surrounding the invasive glands with immune cell infiltration (**Aa**; arrow). NTPDase2 was absent in the desmoplastic stromal cells (**Ba**; arrow). **Ab** is a detail of the tumor-myometrial junctional zone. The lack of NTPDase2 was also patent in the stromal tissue of this zone (**Bb**; * asterisk). Scale bars are 1 mm (**A**,**B**), 200 µm (**Ab**,**Bb**) and 100 µm (**Aa**,**Ba**).

Desmoplasia, frequently reported in invasive endometrial cancer and often presenting abundant inflammatory infiltrate, was observed in the 43 invasive EC tumors studied here. In these tumors, NTPDase2 staining was absent (88.4%) or barely detected (11.6%) in desmoplastic stromal cells wrapping the infiltrating nests with desmoplasia (Figure 4).

#### 3.1.3. NTPDase2^+^/SUSD2^+^ (Sushi Domain Containing 2) Perivascular Cell Population: Endometrial Mesenchymal Stem Cells (eMSCs) Are also Present in ECs

**Figure 5:**
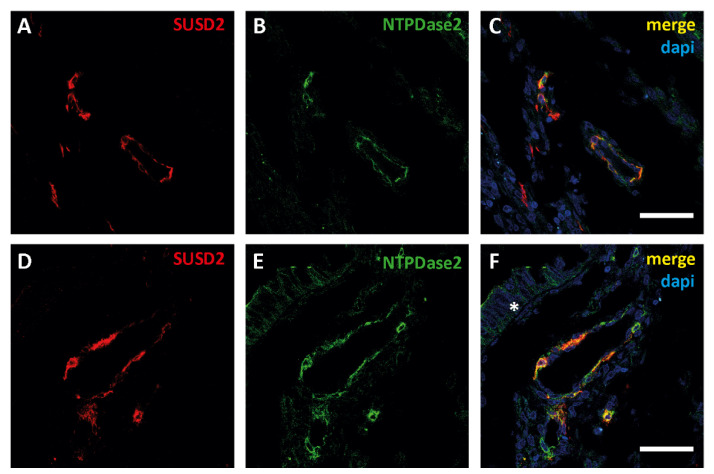
Confocal fluorescence images of different zones of a section of an EC sample with antibodies against sushi domain containing 2 (SUSD2, red, **A**,**D**) and NTPDase2 (green, **B**,**E**). Merged images (yellow, **C**,**F**) show the colocalization of SUSD2 with NTPDase2 in cells surrounding blood vessels in the tumor. Moreover, NTPDase2 was also detected in the tumor cells (**F**, * asterisk). Nuclei were labeled with DAPI (blue, **C**,**F**). Scale bars are 50 µm.

NTPDase2 expression was also observed in a population of cells surrounding some vessels in both the tumor and the myometrium. In the non-pathological endometrium, this population of perivascular eMSCs positive for SUSD2 and NTPDase2 has already been described [7]. The double labelling with anti-SUSD2 and anti-NTPDase2 confirmed the perivascular colocalization of these two proteins in endometrial tumors (Figure 5).

#### 3.1.4. Coexistence of Adenomyosis and Endometrial Polyps with ECs: Comparison of NTPDase2 Expression in Tumor and Non-Tumor Tissues

Adenomyotic lesions, arising from the basal layer of the endometrium, and endometrial polyps, are a good illustration of NTPDase2 expression in the non-tumoral endometrial tissue of patients.

**Figure 6:**
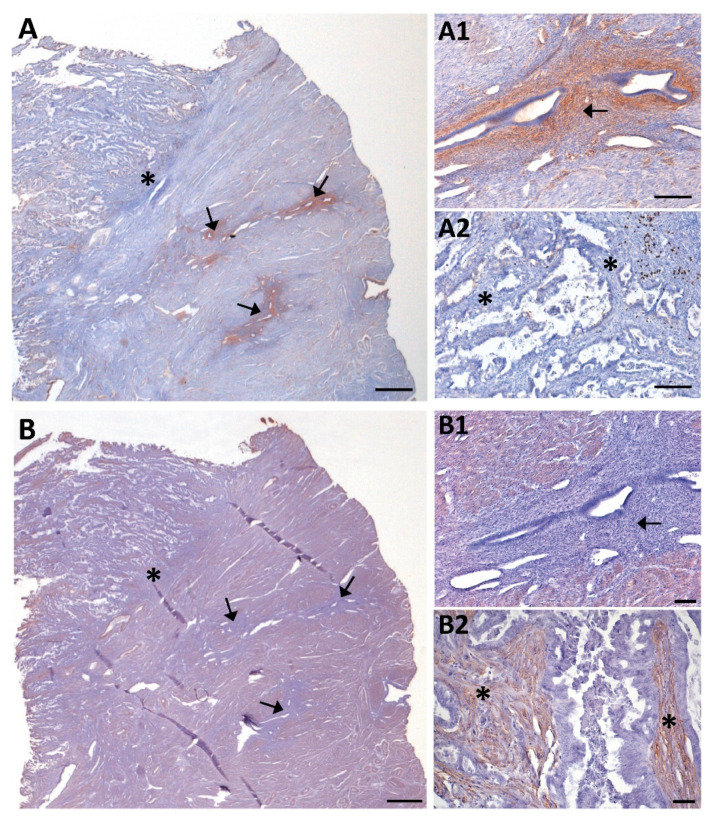
NTPDase2 (**A**) and α-SMA (**B**) immunolabeling of consecutive sections of a case of invasive EC coexistent with adenomyotic lesions (arrows). (**A1**,**A2**) and (**B1**,**B2**) are details of (**A**) and (**B**) respectively. Adenomyosis in the myometrium appeared as non-pathologic endometrial glands surrounded by endometrial stroma expressing NTPDase2 (**A1**; arrow). Conversely, α-SMA was not detected in the endometrial stromal cells of adenomyotic lesions (**B1**; arrow). NTPDase2 was not expressed in the stroma of the tumor (**A2**; * asterisks), while α-SMA was present (**B2**; * asterisks). Scale bars are 1 mm (**A**,**B**), 100 µm (**A1**,**A2**,**B1**) and 50 µm (**B2**).

Adenomyotic lesions were detected in 8 cases of the recruited ECs, 7 being invasive tumors (Supplementary Figure S1). Six of these invasive ECs coexistent with adenomyotic lesions presented expression of NTPDase2 in the endometrial stromal cells of the adenomyotic lesion, but not in the desmoplastic stromal cells located in the invasive front (Figure 6A). In contrast, α-SMA was immunodetected in the desmoplastic stromal cells of invasive front but not in endometrial stromal cells of the adenomyotic lesions (Figure 6B). The only case of invasive tumor that lacked NTPDase2 staining in desmoplastic stromal cells was also devoid of NTPDase2 staining in the stromal and perivascular cells of the adenomyotic lesion.

Additionally, NTPDase2 was detected along the entire length of the cilia of endometrial ciliated cells in adenomyotic lesions (Supplementary Figure S2). We also detected perivascular labeled NTPDase2 in some of these adenomyotic lesions (Supplementary Figure S2).

In the only case in which we found the coexistence of non-invasive EC with adenomyosis, we detected NTPDase2 expression in both the adenomyotic stroma and the stromal cells of the endometrial–myometrial junction.

**Figure 7:**
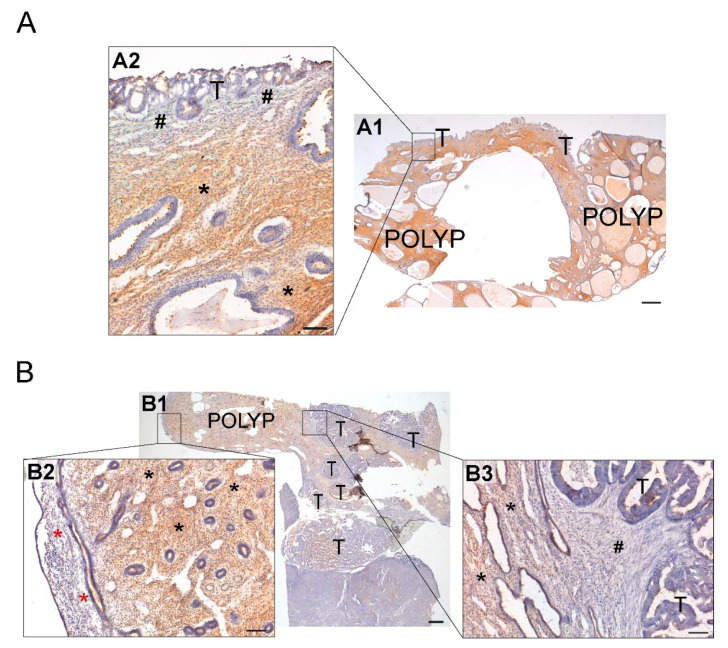
Immunodetection of NTPDase2 in two cases of ECs coexisting with endometrial polyps (endometrial non-tumoral tissue) (**A**,**B**). (**A1**) Invasion of endometrial polyp by serous carcinoma. Invasive tumor cells (T) appeared surrounded by stromal cells lacking NTPDase2 (**A2**; # pound signs), while endometrial stromal cells of endometrial polyp showed NTPDase2 label (**A2**; * asterisks). (**B1**) Invasion of endometrial polyp by grade 1 endometrioid endometrial carcinoma (EEC). Image (**B2**) shows the functional layer of endometrium with a negative stroma for NTPDase2 (* red asterisks). NTPDase2 was immunodetected in the endometrial stroma of endometrial polyp (**B2**,**B3**; * black asterisks). Desmoplastic stroma surrounding invasive endometrial tumor cells did not show NTPDase2 label (**B3**; # pound sign). Scale bars are 1 mm (**A1**,**B1**) and 100 µm (**A2**,**B2**,**B3**). T: tumor cells; POLYP: endometrial polyp.

Finally, we also studied two cases with coexistence of invasive ECs, one serous carcinoma and one EEC (grade 1), with endometrial polyps. NTPDase2 expression in the endometrial polyp glands was limited to the cilia of ciliated cells (as in non-pathological endometrium), while endometrial tumor cells presented an apical expression of NTPDase2 and lacked cilia. As expected, NTPDase2 expression was also detected in stromal cells of endometrial polyps, as described in the basalis of the non-tumoral endometrium. Conversely, the invasive stroma and/or desmoplastic stroma enwrapping invasive tumor glands did not present NTPDase2 labeling. NTPDase2 expression was also detected in some perivascular cells in endometrial polyps. The two analyzed cases are included in Figure 7.

#### 3.1.5. Overall-Survival Probability and Progression-Free Survival: Patients with High Levels of NTPDase2 Show a Better Prognosis for EC

Kaplan–Meier for overall-survival probability and the progression-free survival ratios were calculated for a cohort of patients diagnosed with endometrial carcinoma of endometrium from TCGA. Gene analysis revealed two groups: cases with high and those with low *ENTPD2* expression. We compared the survival probabilities of the two groups (Figure 2C,D).

A significantly increased overall survival probability was observed in the high *ENTPD2* EC group compared with the low *ENTPD2* expression ECs. The log rank analysis showed statistically significant differences with a *p*-value of 0.044, indicating that there was an influence of the *ENTPD2* expression in the prognosis of endometrial carcinoma patients.

Disease-free survival probability was also higher in the high *ENTPD2* expression group. The log rank analysis showed statistically significant differences with a *p*-value of 0.025, suggesting that high *ENTPD2* expression accounts for longer disease-free survival periods.

## 4. Discussion

The purinergic system is the extracellular signaling pathway, mediated by nucleotides and nucleosides, involved in the control of multiple physiological and pathological processes, including proliferation, differentiation, motility, migration, cell death, and immunological response [5,6,10]. These events are essential components of neoplastic transformation, tumor generation, and cancer progression. Therefore, the large number of emerging studies on purinergic signaling in the cancer context is not surprising [24,31]. The study of ectonucleotidases, the specialized nucleotide-hydrolyzing enzymes that regulate the levels of extracellular ATP, has become crucial to our understanding of endometrial homeostasis in health and disease.

EC is the most frequent of the malignant tumors of the female genital tract with an important intertumoral heterogeneity, depending on histological and molecular types, and also intratumoral, due to the different cell components within the same tumor [32,33]. The study of the protein expression profile of EC, including the study of ectonucleotidases, contributes to the improvement of the diagnosis and tumor staging and, in consequence, the clinical management of patients [34].

In the present study we have characterized the expression of NTPDase2, in both the epithelial and the stromal component, of low- and high-grade ECs. NTPDase2 distribution was recently reported in non-pathological endometrium, with expression in three different locations without changes along the cycle or with menopause. Epithelial location was restricted to the cilia of epithelial ciliated cells, while stromal expression exclusively matched the basalis. Finally, NTPDase2 is also a marker of the perivascular SUSD2^+^ eMSC population [7].

In the epithelial component of the tumor, low-grade ECs (grade 1) displayed a similar pattern of NTPDase2 expression to that of the non-tumoral endometrium, exclusively staining the cilia [7]. The presence of cilia in endometrial tumors is not unusual and has been related mostly with better differentiated tumors [35], which is consistent with these results. High-grade tumors (grade 3) changed the pattern of NTPDase2 expression, acquiring a predominant whole-cell staining, which is never observed in the non-tumoral endometrial epithelium. Grade 2 ECs showed an intermediate pattern, with expression of NTPDase2 simultaneously in both locations, reflecting a transitional state. Finally, a number of grade 3 tumors lose NTPDase2 expression. This coincides with the decreased CD73 expression reported in tumor cells of ECs [27]. Our results indicate a possible involvement of NTPDase2 in tumor progression and malignancy in EC, as observed in other cancers. In hepatocellular carcinoma, for example, NTPDase2 promotes tumor growth and the maintenance of myeloid-derived suppressor cells, allowing cancer cells to escape immune surveillance by suppressing T cells in mice [36]. In gliomas, where there is low NTPDase2 activity, the enzyme restores favored tumor progression and spreading in a rat model [37,38].

In terms of the stroma of tumors, the analysis of NTPDase2 expression revealed a relevant finding of importance for diagnosis. NTPDase2 stromal expression, when present, is limited to the tumor stroma at the endometrial–myometrial junction. Importantly, there is loss of expression in correlation with the acquisition of an invasive phenotype, independently of the histological type or even the grade. Therefore, tumors with non-invasive phenotype have NTPDase2^+^ stroma, while tumors that invade the myometrium mostly lose expression. Remarkably, invasive EC samples with non-invasive areas showed NTPDase2 expression, specifically, in these zones.

As mentioned, in the non-pathological endometrium, NTPDase2 has been described as a marker of basalis since its expression is restricted to this layer, without changes along the menstrual cycle. Normal endometrial homeostasis depends on the integrity of the endometrial–myometrial junction, the area of intimate contact between the deepest part of the basalis and the myometrium, and alterations in the endometrial–myometrial junctional zone (e.g., changes in thickness) can lead to disorders such as endometriosis, adenomyosis, and even cancer [39,40,41].

Moreover, tumor invasion involves crosstalk between tumor epithelial and stromal cells [42,43,44,45,46]. Therefore, the loss of stromal NTPDase2 expression at the endometrial–myometrial junction might be involved in modifications of the invasive ability of endometrial tumor cells. A clear example of how stromal NTPDase2 expression can influence the behavior of epithelium is found in liver, under normal conditions. Expression of NTPDase2 by portal fibroblasts inhibits activation of basolateral P2Y receptors expressed by bile duct epithelia, downregulating bile duct proliferation. The loss of expression of NTPDase2 in portal fibroblasts triggers bile ductular hyperproliferation, while there is also a transdifferentiation in portal fibroblasts, which change to myofibroblast-like cells, in biliary fibrosis and cirrhosis [47,48]. In fact, it has been suggested that the loss of NTPDase2 is the earliest event in myofibroblastic transdifferentiation of portal fibroblasts [49]. Based on the results shown here and in accordance with the literature, loss of stromal NTPDase2 expression in tumors, with the acquisition of a myofibroblast-like phenotype, might well be a necessary change to promote myometrial invasion [50,51,52]. In line with this, we confirmed that tumor stromal cells acquire a myofibroblast-like phenotype (α-SMA^+^) exclusively in invasive ECs. This α-SMA staining in stromal cells accompanying the invasive foci in the myometrium matched the lack of NTPDase2 expression in these cells. These results support the hypothesis of a loss of NTPDase2 as an early event in the transdifferentiation to a myofibroblast-like phenotype.

Several studies have examined the presence of myofibroblast-like cells in stroma of invasive tumors [51,52,53,54]. It has been suggested that there exist two heterogenic populations of cancer-associated fibroblasts (CAFs) in tumoral stroma: type I (without expression of α-SMA), which limits tumor growth [55], and type II (with upregulated expression of α-SMA and vimentin), which promotes angiogenesis and metastasis of the tumor [46,55]. Based on the results shown here, it seems that type I CAFs are mainly present in non-invasive ECs, while the stroma of invasive ECs is mainly composed of type II CAFs (α-SMA^+^). Previous studies in EC established that type II CAFs (α-SMA^+^) trigger proliferation, migration, and invasion of endometrial tumoral cells as well as in in vivo tumorigenesis [56,57]. Further studies are needed to elucidate the precise role of NTPDase2 in EC progression and invasiveness.

Our results point to the usefulness of NTPDase2 as a marker of tumor invasion in EC, especially in cases in which EC coexists with other endometrial disorders, such as adenomyosis, which occasionally hampers the assessment of parameters of tumor invasion [58]. When a myometrial invasion statement might be challenging due to the presence of adenomyosis foci [59,60], the use of NTPDase2 expression might help to overcome this limitation, since adenomyosis retains the expression while invasive (and desmoplastic) tumor areas of the sample do not. Other markers have been studied with the purpose of differentially labeling invasive and non-invasive tumor stroma and adenomyotic stroma. CD10 is a broadly used marker of endometrial stroma but does not distinguish between different invasive phenotypes [61,62,63,64]. More recently, interferon-induced transmembrane protein 1 (IFITM1/CD225) has been identified as a stromal marker differentially labeling the stroma depending on the invasive phenotype [64]. The expression pattern of IFITM1 is very similar to that presented here for NTPDase2 although IFITM1 labels the whole stroma.

In addition, our results of NTPDase2 expression in adenomyotic lesions and endometrial polyps match our previously published description of NTPDase2 expression in endometrial samples without cancer pathology, where NTPDase2 was found in the cilia of ciliated cells and in stromal cells [7]. Moreover, we show here that the perivascular SUSD2^+^/NTPDase2^+^ eMSCs population is also present in ECs.

Finally, the conclusions of the study of the *ENTPD2* gene expression profile in 527 ECs included in TCGA were in line with the analysis at the protein level, specially at the stroma. There are two groups with clinical impact in relation to high or low levels of *ENTPD2* expression. High *ENTPD2* levels in EC patients significantly correlated with a better prognosis and longer-disease free survival, displaying higher OS and PFS ratios. This coincides with the decrease in protein expression in the stroma of invasive tumors, with a worse prognosis.

**Figure 8:**
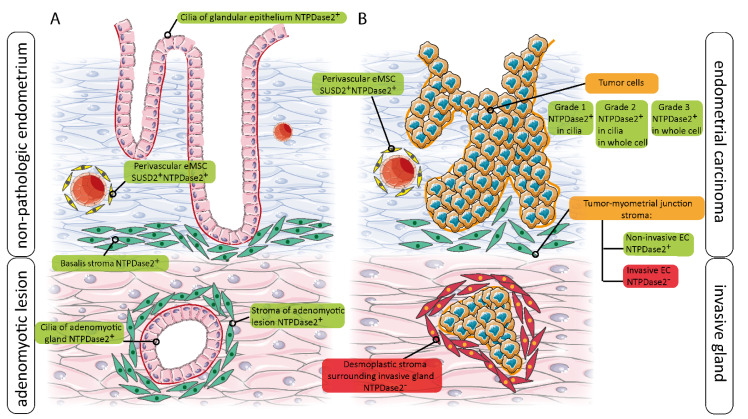
Schematic representation of NTPDase2 changes of expression in relation to non-tumoral endometrium (non-pathologic, and adenomyotic tissue) (**A**) and in the invasive and non-invasive ECs (**B**).

A graphical summary is shown in Figure 8, where the distribution and expression changes of NTPDase2 in non-pathological, adenomyotic, and tumor endometria are described. This summary clearly shows that stem cell markers, such as NTPDase2, can be expressed in other cell types.

## 5. Conclusions

This is the first report of NTPDase2 expression in ECs, with changes matching different invasive phenotypes. The analysis of the NTPDase2 expression at the endometrial–myometrial junction evidences its potential use as a marker to differentiate between non-invasive, NTPDase2^+^ stroma, and invasive, desmoplastic NTPDase2^-^ stroma. Adenomyosis foci and endometrial polyps are also clearly NTPDase2^+^. The loss of NTPDase2 coinciding with acquisition of α-SMA expression in invasive stromal cells opens up a promising new field of study in ECs.

Furthermore, we confirmed that the SUSD2^+^/NTPDase2^+^ eMSC population is retained in EC.

The cell pathways in which NTPDase2 is involved in EC are in need of deciphering. Its activity might influence the final balance of available nucleotides in the tissue microenvironment, as in the case of proinflammatory extracellular ATP in endometriosis [7].

## Figures and Tables

**Table 1:** Descriptive statistics of endometrial tumor samples.

EC Histologic Types		Number of Total Cases (*n*)	Cases without Myometrial Infiltration	Cases with Myometrial Infiltration		Adenomyotic Lesions
				with Desmoplastic Reaction	without Desmoplastic Reaction	
Endometrioid carcinoma	Grade 1	20 *	5	12 *	3	4
	Grade 2	10	1	8	1	0
	Grade 3	17	2	14	1	2
Serous carcinoma		5 *	0	5 *	0	1
Carcinosarcoma		3	1	2	0	1
Mixed carcinoma		2	0	2	0	0
Total		57	9	43	5	8

* Including a case of endometrial carcinoma (EC) in endometrial polyp.

## Data Availability

Not applicable.

## Supplementary Materials

The following are available online at https://www.mdpi.com/article/10.3390/jpm11050331/s1, Supplementary Figure S1: Haematoxylin and eosin staining in a case of invasive EC coexistent with adenomyosis, Supplementary Figure S2: NTPDase2 immunodetection in adenomyotic lesion from a case of EC coexistent with adenomyosis.
