# Supplementary material for: Characterization of the Endometrial MSC Marker Ectonucleoside Triphosphate Diphosphohydrolase-2 (NTPDase2/CD39L1) in Low- and High-Grade Endometrial Carcinomas: Loss of Stromal Expression in the Invasive Phenotypes
Source: J Pers Med. 2021 Apr 22;11(5):331. doi: 10.3390/jpm11050331 (PMC8146812; doi:10.3390/jpm11050331)
Supplement: Supplementary file 1 [file jpm-11-00331-s001.zip › jpm-1153371-supplementary.pdf]

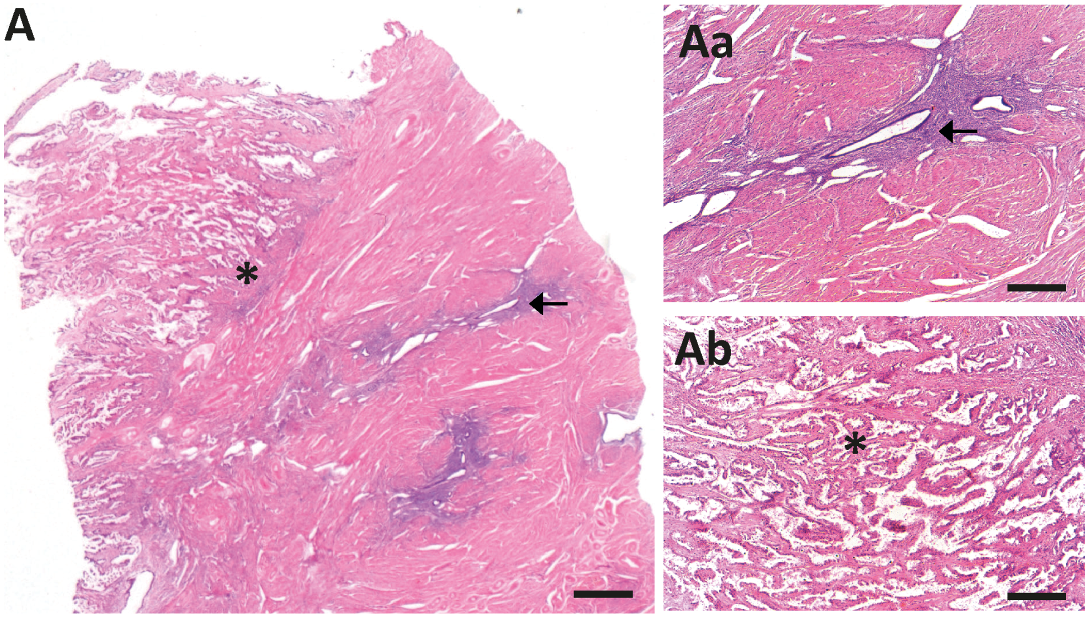

Supplementary Figure 1. Haematoxylin and eosin staining in a case of invasive EC coexistent with adenomyosis. Adenomyotic lesions are composed of endometrial glands and highly cellular stroma, and for this reason, these foci appeared mainly dyed with haematoxylin (Aa; arrow). Conversely, tumor stroma is less cellular and more fibrotic (desmoplastic), with greater affinity for eosin (Ab; asterisk). Scale bars are 1 mm (A) and 100 μm (Aa,Ab).

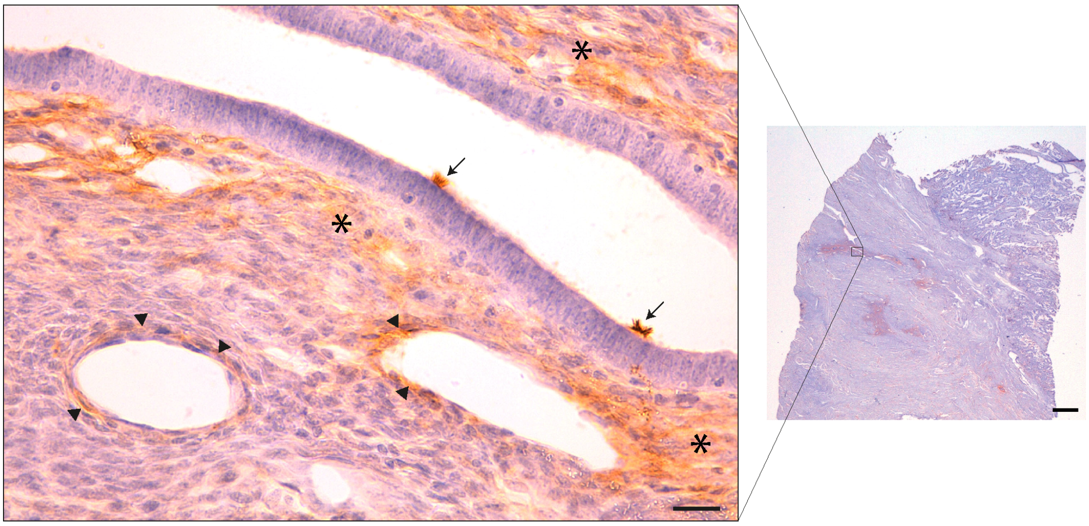

Supplementary Figure 2. NTPDase2 immunodetection in adenomyotic lesion from a case of EC coexistent with adenomyosis. NTPDase2 label is present in the cilia of endometrial ciliated cells (arrows), in the stroma (asterisks), and in perivascular cells of adenomyotic lesion (arrowheads). Scale bars are 1 mm and 20 μm (detail).
